# An Autonomous Robot-Aided Auditing Scheme for Floor Cleaning

**DOI:** 10.3390/s21134332

**Published:** 2021-06-24

**Authors:** Thejus Pathmakumar, Manivannan Kalimuthu, Mohan Rajesh Elara, Balakrishnan Ramalingam

**Affiliations:** Engineering Product Development Pillar, Singapore University of Technology and Design (SUTD), Singapore 487372, Singapore; pathmakumar_thejus@mymail.sutd.edu.sg (T.P.); manivannan_kalimuthu@mymail.sutd.edu.sg (M.K.); rajeshelara@sutd.edu.sg (M.R.E.)

**Keywords:** autonomous cleaning audit, cleaning benchmark, audit robot, dirt driven exploration

## Abstract

Cleaning is an important factor in most aspects of our day-to-day life. This research work brings a solution to the fundamental question of “How clean is clean” by introducing a novel framework for auditing the cleanliness of built infrastructure using mobile robots. The proposed system presents a strategy for assessing the quality of cleaning in a given area and a novel exploration strategy that facilitates the auditing in a given location by a mobile robot. An audit sensor that works by the “touch and inspect” analogy that assigns an audit score corresponds to its area of inspection has been developed. A vision-based dirt-probability-driven exploration is proposed to empower a mobile robot with an audit sensor on-board to perform auditing tasks effectively. The quality of cleaning is quantified using a dirt density map representing location-wise audit scores, dirt distribution pattern obtained by kernel density estimation, and cleaning benchmark score representing the extent of cleanliness. The framework is realized in an in-house developed audit robot to perform the cleaning audit in indoor and semi-outdoor environments. The proposed method is validated by experiment trials to estimate the cleanliness in five different locations using the developed audit sensor and dirt-probability-driven exploration.

## 1. Introduction

The impact of cleaning and cleanliness can span from an individual’s tiny social space to a nation [1]. It is reported that, the cleaning related industry and services market is valued more than 292.6 Billion USD in 2019, and it is expanding steeply with a growth rate above 5% annually around the globe [2,3]. The importance of cleaning acts as a pull factor for the entry of newfangled technologies into the domestic and professional cleaning services, targeting the improvement of the quality and productivity of the cleaning. This includes effective disinfection strategies and automation of cleaning process using robots [4,5,6].

A large volume of research work is reported under cleaning robots for the past ten years. For instance, the development of a terminal floor cleaning robot is discussed in [7], where authors emphasize on robotic ultra-violet disinfection strategy for eliminating pathogens. Lee et al. reported a study on the mechanism and control of the robot for glass façads cleaning [8]. The work mentioned in [9,10] discusses the successful usage of re-configurable mechanisms for improving the area coverage of a robot by adapting various morphology concerning the operational environment. Liu et al. discusses sensor-based complete coverage path planning for cleaning robots, where the reported works claim the effectiveness in most work-spaces [11]. The research work mentioned in [12] discusses a strategy for choosing an optimal footprint for efficient cleaning task execution by a ship hull cleaning robot. The research focuses on robot-aided cleaning is centered around the invention of novel mechanisms for space accessibility, methods, and tools for energy efficient cleaning, optimal path planning, and smart and intelligent autonomous cleaning behavior for robots. The cleaning performance by the robots is assessed mostly by the area coverage of the robot under the notion that if a robot covers an area, it is assumed to be cleaned. The verification of the cleaning quality remains as archetypal visual inspection. Hence, an effective method for inspecting the finer details of cleaning quality is essential to assess the cleaning performance of the robot. A multitude of methods are reported to perform a cleaning process efficiently. However, primitive focus were given to studying the extend of cleanliness of an area after a cleaning process. The cleaning auditing process is done either using visual inspection methods or by microbial analysis [13,14,15]. One among the attempts for evaluating the cleaning performance is ATP (adenosine triphosphate) bio-luminescence method and benchmarking in relative light units (RLU) [16,17,18]. The applicability of ATP bio-luminescence methods are confined to hospitals and food handling environments where pathogenic infestation control is critical. Nevertheless, cleaning is indispensable in a broad spectrum of the domain, including every industrial and domestic setting. Hence, the cleaning auditing strategies should be scalable beyond microbial analysis for a limited environment similar to hospitals and food processing industries.

Robot aided cleaning is a field that is advancing by leveraging cutting-edge technologies like artificial intelligence, sensing, energy-efficient systems, etc. [19,20,21,22]. An effort to bridge the research gap in estimating the quality of cleaning and formulating a strategy to execute it using robots can bring a solution to the paradox—“how clean is clean”. Even though a strong precedence for robot-aided cleaning assessment is missing, a superficial research effort towards detecting the extent of cleanliness of a surface is evident. Especially to estimate the number of dirt particles accumulated on a surface. For instance, a vision-based dirt detection method is discussed in [23] for performing selective area coverage by a re-configurable robot. Grünauer et al. proposed an unsupervised learning-based dirt detection strategy for automated floor cleaning using robots [24]. The research work mentioned in [25] utilized neural networks for the development of locating the dirt for autonomous cleaning robot.

In this article, we propose a novel strategy to assess the extent of cleanliness using an autonomous audit robot. The method of auditing is formulated using artificial intelligence-based vision system together with a new dirt-probability-driven exploration strategy and a sensor module for auditing by extracting dirt from a given area. As per the proposed framework, the robot can trans-locate inside a built infrastructure carrying the audit sensor to analyze the cleanliness of the floor. Upon completion of the auditing, the robot provides a dirt density map, and an estimate of dirt distribution corresponds to the location. The insight of cleaning quality and dirt accumulation pattern reported by the audit robot opens up the opportunity to benchmark either human cleaning or automated cleaning in an effective way. The proposed framework is simple and interoperable with a conventional autonomous mobile robot capable of performing Simultaneous Localization and Mapping (SLAM). This article is organised as Section 2 detailing the general objective of this study followed by the overview of the proposed system in the Section 3, working principle and development of audit sensor in Section 4. The methods to integrate the framework and exploration strategy for the robot is detailed in Section 5 and Section 6. The validation of the system through experiment results are given in Section 7, followed by the conclusion of our findings and future work in the Section 8.

## 2. Objective

This study is designed to put together a framework to quantify the extend of cleanliness of a built-infrastructure using an autonomous robot. This general objective is subdivided into three components:

1. Develop an audit sensor capable of analysing the extent of cleanliness at a given point on the floor.
2. Integrate the audit sensor in an in-house developed mobile robot, and formulate an exploration strategy for auditing an area.
3. Experimentally determine the cleanliness benchmark score of the audit area using the in-house developed mobile robot.

## 3. Cleaning Audit Framework Outline

**Figure 1:**
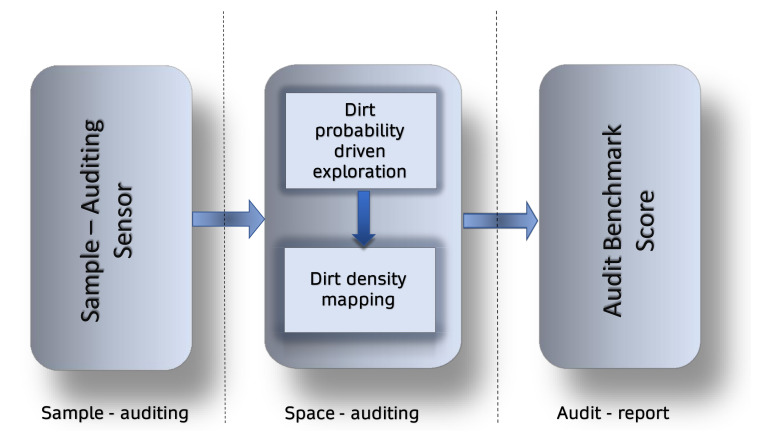
Processes involved in an auditing and benchmark framework for cleaning.

An outline of a simple auditing and benchmark framework is illustrated in Figure 1. An autonomous cleaning audit is comprised of two auditing processes, sample auditing and space auditing. Sample-auditing is the assessment of dirt accumulation in a given point. Space-auditing is the repeated sample-auditing in the entire space to obtain an overall information of dirt distribution. The sample auditing is realized using an auditing sensor that assigns an auditing score for a given point under inspection. The space-auditing is realised with a mobile robot which is capable of exploring the territory with an auditing sensor on-board. It is not practical to do the sample auditing in a finer resolution for a vast area. Hence, the exploration algorithm of a robot has to be smart by reducing the sample auditing space with the help of specific heuristics about the probabilities to find a dirt. This is realized by a dirt probability driven exploration, where the exploration is driven by heuristics regarding the probability for finding dirt. Upon completion of exploration, the robot provides an audit report comprised of a dirt density map and a cleaning benchmark score. The proposed strategy for cleaning auditing is simple to integrate onto an existing autonomous robot.

## 4. Audit Sensor

**Figure 2:**
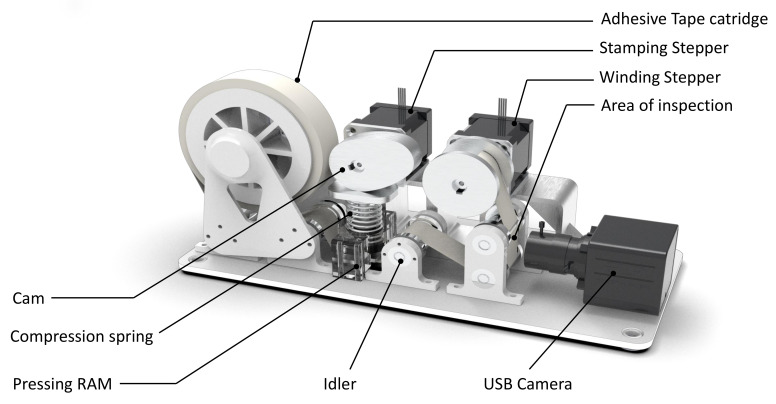
An overview of sample auditing sensors and its major components.

Figure 2 shows the diagram of the sample auditing sensor and components associated with it. An auditing sensor provides information of the extend of cleanliness of a sample area. In the case of floor cleaning, the sensor should detect the amount of dirt distribution in a sample point (a small section of area) on the floor. The sensor module consists of an embedded MPU for handling the computation tasks and a pair of bipolar stepper motors to enable the sample collection action. The sensor is also equipped with a USB camera with a fixed focal length to capture the close-range picture of collected dust samples and an MCU unit that does low-level hardware integration and actuator control. The sensor can be interfaced with the robot either by USB or by Ethernet. The sensor takes the sampling instruction from the robot and provides an “audit score“ that represents the amount of dirt accumulation at the point.

### Working Principle

Stepper actuates a pressing RAM vertically down and the adhesive tape makes contact with the floor, resulting in the adhesion of dirt particles on it. The adhesive tape is moved under the field of view of a camera for computer vision-based auditing. Upon receiving a sample collection request from the robot, the winding stepper moves 4 cm of adhesive tape from the roll. The adhesive tape on the sensor can be replenished with a new one after repeated sampling collection. A sample point is considered as a 30 mm × 20 mm rectangular patch on the floor.

Certain parameters can be used to estimate the cleanliness of a region by analyzing the collected sample. For instance, some of the cleanliness determining parameters could be the number of dirt particles concentrated in the sample or the changes in color and texture due to dirt accumulation. If cleanliness is defined at a microscopic level, microbial infestation could be a parameter that defines cleanliness. For a vision-based sample auditing approach, the sample audit score can be computed based on the above-mentioned cleanliness determining parameters extracted from the sample image captured by the camera after the dust extraction from the floor. A weighted average of the extracted parameters gives the sample audit score. The Equation (1) computes the sample audit score from *n* distinct parameters that are extracted from sample auditing, where $P_{i}$ and $w_{i}$ are ith parameter and weight corresponding to it.

(1) $$S_{n}=\frac{\sum_{i=1}^{n}w_{i}P_{i}}{\sum_{i=1}^{n}w_{i}}$$

**Figure 3:**
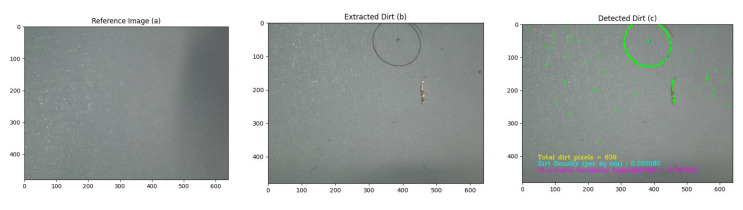
Reference image taken by the sensor (**a**), dirt extracted from floor by the audit sensor (**b**), green region represents the dirt detected (**c**).

In a typical floor cleaning auditing scenario, sum of absolute differences (SAD) and the Mean Structural Similarity Index Measure (MSSIM) are two suitable parameters for computing audit scores. In the domain of computer vision, SAD gives a measure of similarity between two images. It is calculated by taking the absolute difference between each pixel in the test image and the corresponding pixel in the reference image [26,27]. In the given scenario, the test image and reference images are taken as the image captured after dust extraction, from the surface of adhesive tape, and the image captured before dust extraction. These inferred differences between the test image and reference image are summed up to form a similarity image, which can be mathematically represented as in Equation (2).

(2) $$I_{Similarity}\left(t\right)=\sum_{i=0}^{M}\left|I_{Test}\left(i\right)-I_{Ref}\left(i\right)\right|$$

where, $I_{Similarity}$, $I_{Test}$, $I_{Ref},$ and *M* are similarity image, test image, reference image and resolution respectively. A threshold range is applied on the similarity image, and a binary image (1 corresponds to dirt pixels, 0 corresponds to non-dirt pixels) is obtained, which holds the information of possible pixels representing dirt (Figure 3). From the binary image, dirt density ρ can be computed using Equation (3).

(3) $$\rho =\frac{No\;of\;dirt\;pixels}{total\;no\;of\;pixels}$$

Besides the dirt density from SAD, the structural similarity index of the image is calculated to determine extent of dirt particles populated on the adhesive tape surface after the dust lifting. Structural Similarity Index is one of the popular methods to compute the quality assessment between two images [28,29]. The approach mentioned above has been taken under the notion that the similarity with the reference image (clean image) will be altered when dust particles appear on the captured test image. The structural similarity index is comprised of luminance, contrast, and structural similarity features from an image. A Mean Structural Similarity Index (MSSIM) is an average value of similarity indices taken across multiple sections of an image (Equation (4)).

(4) $$MSSIM=\frac{1}{M}\sum_{i=1}^{M}SSIM\left(x_{i},y_{i}\right)$$

where, SSIM is the Structural Similarity Index Measure calculated at every pixel on the image [28]. The MSSIM values will be lower for an image with fewer dirt pixels on it (less similar to the reference image) compared to an image that captured no dirt (more similar to the reference image). The MSSIM is range of values in the interval [−1,+1]. Since the parameter $P_{i}$ in Equation (1) only takes in values in the range [0,+1], $MSSIM_{Mapped}$ is calculated to map the MSSIM value to the desired interval [0,+1] using Equation (5).

(5) $$MSSIM_{Mapped}=\frac{1-MSSIM}{2}$$

Since ρ gives the dirt density, the significance of ρ is higher when dirtiness is caused by dust particles. Similarly, the significance of $MSSIM_{Mapped}$ is higher when dirtiness is caused by colored or colorless stains on the floor. Since both factors are equally important, equal weights are given for both for the floor cleaning scenario. The sample audit score $S_{2}$ (n=2 and $w_{1}=w_{2}=1$) can be calculated by:

(6) $$S_{2}=\frac{\rho +MSSIM_{Mapped}}{2}$$

The audit score calculation is done on the embedded MPU on the sensor. The dust extraction and audit score calculations are triggered upon a request from the robot, and the audit score is updated back to the robot.

## 5. Audit Robot and Framework Integration

**Figure 4:**
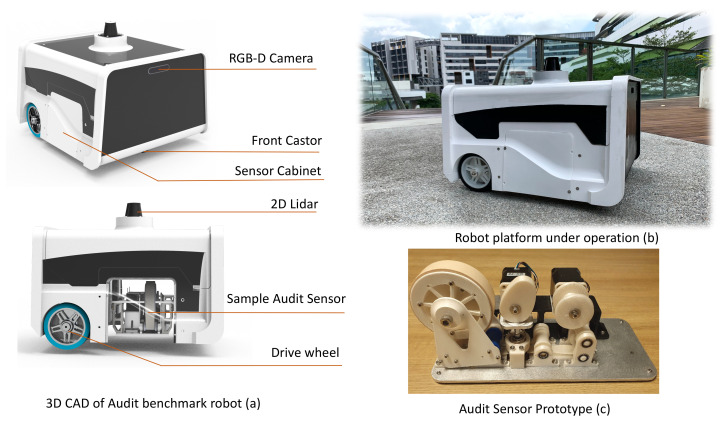
CAD diagram of robot (**a**), Prototype robot under operation in a food court (**b**), Sample audit sensor prototype (**c**).

An autonomous mobile robot equipped with an auditing sensor is the key facilitator for the space-auditing process in the proposed cleaning auditing framework. Figure 4 shows the developed audit robot capable of performing autonomous navigation and mapping. The robot with an audit sensor on-board explores its area of operation using dirt-probability-driven exploration, and an audit report is generated. The audit report comprised of dirt density map and dirt distribution pattern generated from the information from robot’s audit sensor and 2D map.

### Audit-Robot Architecture

**Figure 5:**
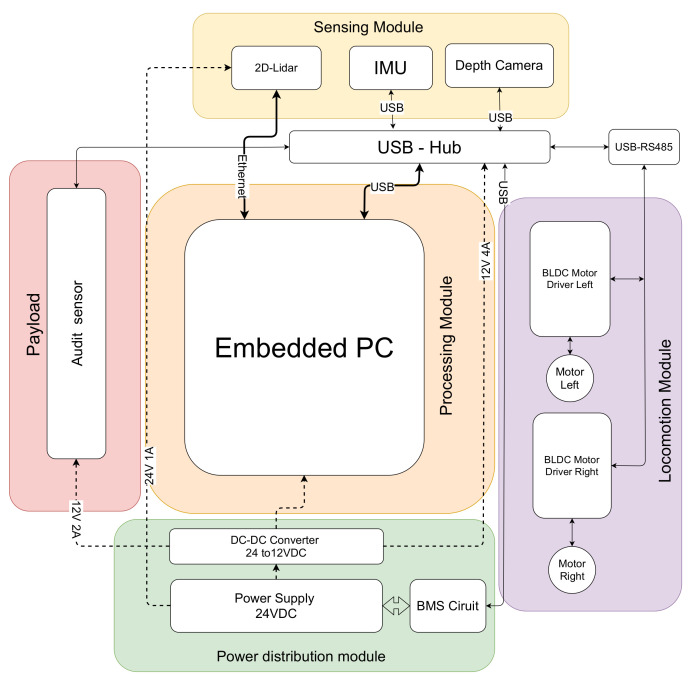
Block diagram of system architecture of Audit-Robot with audit sensor payload.

The system architecture of the audit robot is shown in Figure 5. The robot platform has a differential drive wheel configuration with three-point of contact. A pair of brushless DC (BLDC) motors (left and right) is the primary drive mechanism of the robot. Two BLDC motor drivers control the speed, acceleration, and direction of rotation of each motor. The BLDC motor drivers do a closed-loop velocity control of the motors and provide instant velocity feedback. The motor driver is commanded over MODBUS communication protocol implemented over RS485 [30]. The RS485 communication bus of the left and right motor drivers are connected in a daisy chain fashion. A 24VDC $LiFePO_{4}$ battery powers the systems and subsystems of the robot. A 2D LIDAR (Sick TIM 581) and depth camera (Intel Realsense 435i [31]) sensor provide real-time information of the surroundings for perception. The Realsense D435i have maximum resolution 1920 × 1080 with a field of view of 87deg × 58deg A 9-DoF (Vectornav VN100 IMU) and the odometry information from the robot wheels provide the necessary information for dead-reckoning. The software packages for the robot perception, localization, and navigation are implemented over ROS middleware. An Embedded computer with an intel core i7 processor with Ubuntu 20.04 operating system is used to implement the software nodes and low-level drivers. The LiDAR is interfaced with the Embedded PC using Ethernet. The BLDC Motor drivers and IMU sensor are connected to the embedded PC by a USB-RS485 converter.

Besides the sensors for navigation, the robot carries the prototyped audit sensor. An NVIDIA Jetson NX has been used as the embedded MPU for the sensor. The electronics part of the sensor is positioned in the robot. NEMA 17 bi-polar stepper motors are used for enabling sample collection action. A TB6600 stepper motor driver is used for micro-step control of the stepper motor. The sample sensor module is interfaced with the embedded PC of the robot via Ethernet. The power requirement for the sensor is supplied from the robot’s battery.

## 6. Exploration Strategy

This section explains the dirt probability-driven exploration and path-planning strategies that facilitate the cleaning auditing by the audit robot. The robot should set an exploration out with the objective of covering the maximum locations possible and generate a map by SLAM. There are many auto-exploration approaches that are used in mobile robots, such as patrol robots, inspection robots, rescue robots etc. [32,33,34]. Frontier exploration is one of the popular exploration methods in single or multi-robot systems, where the destination pose of the robot is decided by the frontiers in a grid map [35,36,37]. However, for the audit robot, the objective of the exploration aims not only to visit all the locations for mapping but also to cover the most probable dirt regions for sample collection. This is made possible by modifying the frontier exploration strategy. The pseudo-code for modified frontier exploration is given in the Algorithm 1.
**Algorithm 1** Pseudo-code of the exploration strategy with modified frontier exploration algorithm1:F⇐checkFrontier()2:**for** Every points in *F* **do**3:    **if** *F* is a connected frontier **then**4:        $F_{connected}⇐F_{i}$5:    **end if**6:**end for**7:**for** Every points in $F_{connected}$ **do**8:    $C^{open}⇐$ compute centeroid of $F_{connected}$9:    Arrange $C^{open}$ in ascending order of distance from robot pose10:**end for**11:**for**$C^{open}$ is not empty **do**12:    $destination\_pose⇐F_{i}^{open}$13:    Check for Probable Dirt Points.14:    **if** Probable Dirt Points **then**15:        Transform interest point to global frame assign to $IP^{open}$16:        Arrange $DP^{open}$ in ascending order of distance from robot pose17:        **for** Every points in $DP^{open}$ **do**18:           Transform interest point to global frame19:           Generate sample points *S*20:           Select sample points diagonally and insert to SP21:           **if** Selected sample point not in obstacle **then**22:               Insert sample point to SP23:           **end if**24:           **for** Every points in SP **do**25:               Navigate the robot to $SP_{i}$26:           **end for**27:           $DP_{i}^{closed}⇐DP_{i}^{open}$28:        **end for**29:    **end if**30:    Navigate the robot to $C_{i}^{open}$31:    $C_{i}^{closed}⇐C_{i}^{open}$32:    Initiate a $360^{\deg}$ sweep33:    F⇐checkFrontier()34:**end for**

Typically an occupancy grid of robot possess three values. Obstacle region, free space and unexplored region. A frontier is defined as the boundary between a free space and an unexplored region in the occupancy grid. Frontiers are formed either when the boundary is beyond the sensor’s field of view or mapping at the particular territory is incomplete. A perfectly mapped region will be devoid of frontiers in its occupancy grid representation. The frontier exploration directs the robot to navigate towards the frontier and explore repeatedly and extend the boundaries of the map till no new frontiers are left to explore. A frontier can be identified by running a Breadth-First Search (BFS) across the entire grid map. The identified frontier points are pushed to a list *F*. From the frontier points on the data container, connected frontier points above minimum size are identified $F_{connected}$. From the $F_{connected}$, identified frontiers centroid are pushed to an another list in the increasing order of euclidean distance between the robot’s current location provided by the SLAM algorithm $C^{open}$. In the classical frontier exploration strategy, robot navigates to frontier centroids in the list by First In First Out (FIFO) fashion. However, this step has been re-defined in the proposed algorithm for auditing benchmark exploration. Before the algorithm sets the robot’s way-point to a frontier centroid $C^{open}$, it will look for a detected probable dirt region in its field of view by the dirt region locator algorithm using semantic segmentation and periodic pattern detection. If a probable dirt region is detected in its filed of view, the centeroids of probable dirt regions are pushed to a list $DP^{open}$ (probable dirt region open list) in ascending order of euclidean distance from the current robot pose.

**Figure 6:**
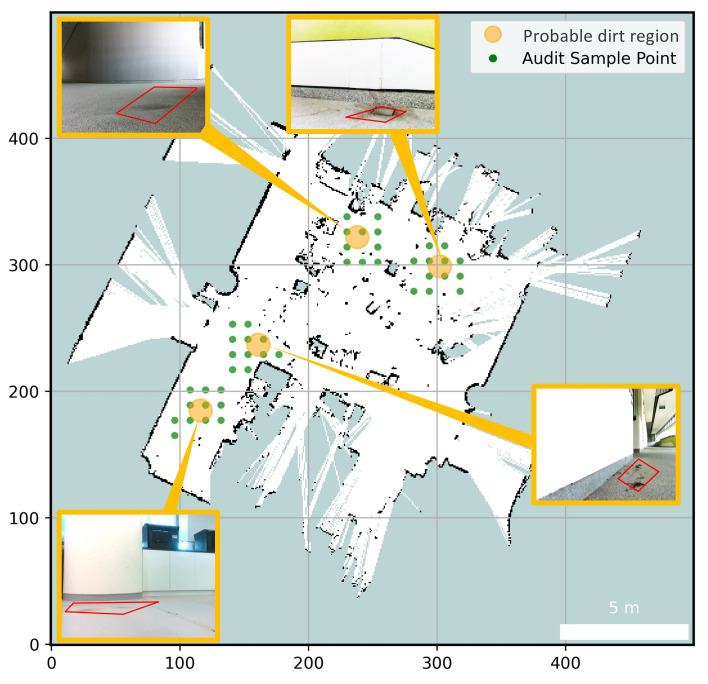
Sample points and probable dirt region identified by the robot.

A breadth first search (BFS) is executed in the list $DP^{open}$ and a square region around the probable dirt region is selected. The selected square region is sampled uniformly to obtain the set of sample points corresponding to $DP^{open}$. The sample points are selected and arranged in a zig-zag fashion (diagonal selection). If the selected sample points are not on an obstacle in the map, the selected sample points are added to a list SP.The robot navigates to every points in SP. While navigating, robot takes the audit samples in every sample points. Once the robot covers all the sample points SP associated with a probable dirt region $DP_{i}^{open}$, it is pushed to the closed list $DP^{closed}$. The robot never generates sample points around probable dirt region in the $DP^{closed}$. Robot continues navigating and generating sample points till every element in $DP^{open}$ is moved to the $DP^{closed}$. When $DP^{open}$ is an empty container, the destination is switched to $C_{i}^{open}$. Once robot reaches a point in $C_{i}^{open}$, $C_{i}^{open}$ is appended to $C_{i}^{closed}$ and a $360^{\deg}$ sweep is performed to scan for next frontiers. This whole cycle is continued till $C^{open}$ becomes an empty list. The Figure 6 illustrates the dirt probability driven exploration strategy, identified probable dirt region and sample points associated it.

**Figure 7:**
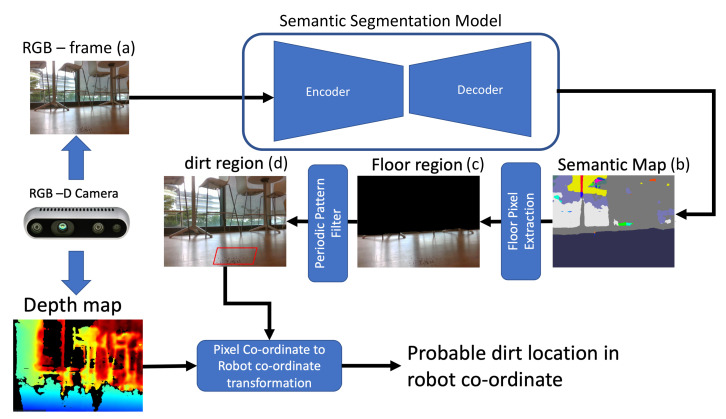
RGB image frame taken from (**a**), segmented output (**b**), extracted floor pixels (**c**), Most probable dirt region (**d**).

The navigation for the robot is done by $A^{*}$ navigation algorithm [38] accompanied by a Dynamic Window Approach (DWA) path following algorithm [39]. The combined usage of $A^{*}$ and DWA allows the robot to move from one point to another without a collision. The position of the robot in the global co-ordinate is provided by the G-Mapping SLAM algorithm [40]. Figure 7 shows the process flow of the probable dirt region identification using semantic segmentation and periodic pattern filter [23]. Major steps are involved in identifying probable dirt location in the field of view of the sensors are:

1. Capture the RGB frame and aligned depth map of the environment;
2. Perform a semantic segmentation and extract the floor region;
3. Extract the dirt region using periodic pattern filter applied on floor region;
4. Using depth map, transform the centroid of dirt region from pixel-coordinate to robot co-ordinate;

The centroid of the identified dirt region on robot co-ordinate is considered as the probable dirt location. The implementation of semantic segmentation and periodic pattern filter are detailed below.

### 6.1. Semantic Segmentation

The semantic segmentation is carried out using ResNet50 [41] and Pyramid Pooling Module (PPM) encoder-decoder method trained on ADE20K indoor dataset [42,43,44,45]. The mask corresponding to the class “floor“ has been extracted from the semantic segmentation output. Using the mask, the pixels corresponding to the floor has been extracted and a periodic pattern suppression algorithm has been executed to identify the most probable dirt region. From the depth image corresponding to the captured RGB frame, the 3D point is located on robot co-ordinate.

### 6.2. Periodic Pattern Filter

The outcome of semantic segmentation gives the pixels corresponding to the floor. This image holds two information, one is the floor pattern and second one is the dirt particles present on the floor. Periodic pattern filter is applied on the largest possible rectangular on the extracted floor region. Periodic pattern filter detects the regular patterns occurring on an image, these periodic patterns are floor texture on the captured image [23].

1. Step 1: Transform the image to a frequency domain using 2D Fast Fourier Transform
  (7) $$F\left(u,v\right)=\sum_{m=0}^{m-1}\sum_{n=0}^{n-1}f\left(i,j\right)\times e^{-i\times 2\pi \times (\frac{u\times i}{m}+\frac{v\times j}{n})}$$
2. Step 2: The periodic pattern filter *H* is determined by applying an FFT shift operation in the input image F(u,v)
  (8) $$\hat{f}\left(u,v\right)=\hat{f}\left(\left[F\left(u,v\right)\right]\right)$$
3. Step 3: Choose the maximum log components from $\hat{f}\left(u,v\right)$
  (9) $$H\left(u,v\right)=log(\hat{f}\left(\left[F\left(u,v\right)\right]\right))$$
  (10) $$H_{max}\left(u,v\right)=max\left(log\left(\hat{f}\left(\left[F\left(u,v\right)\right]\right)\right)\right)$$
4. Step 4: Suppress the frequency components less than $H_{max}$
  (11) $$F^{\prime }\left(u,v\right)=\left\{\begin{array}{cc} F(u,v), & ifH\left(u,v\right)>H_{max}\left(u,v\right)\\ 0, & \mathrm{otherwise} \end{array}\right.$$

An inverse FFT brings back the $F^{\prime }\left(u,v\right)$ back to time domain $F^{\prime }\left(i,j\right)$. The dirt accumulate location is appeared as blobs. A convex hull algorithm is applied to get the boundary of probable dirt location. The centeroid of the detected boundary is considered as the probable dirt location. Each image captured by the depth camera have RGB data in pixel coordinate and depth data (in meters) associated with it. Hence any point in the captured image by the depth camera can be projected to 3D coordinate space. The relation between pixel-coordinate and 3D real-world coordinate systems is given by the intrinsic camera parameters. The intrinsic camera parameters and 3D projection are provided by Intel Realsense SDK associated with the depth camera. The depth corresponds to the centroid of the probable dirt location is identified from the depth map and transformed from pixel coordinates to 3D coordinates to get the probable dirt region in the robot coordinate.

After an sample auditing and space auditing, following parameters are computed to determine the extent of cleanliness of the region:

1. Dirt density map: A grid-map with audit scores are labeled to its corresponding location. The significance of dirt density map is to visualize the sample-level details of dirt accumulation corresponds to a region.
2. Dirt distribution map: Dirt distribution map is a surface plot that shows the probability density function (PDF) of dirt accumulation. PDF of dirt is modelled using bi-variate Kernel Density Estimation (KDE) over the sample locations [46,47].
3. Total audit score (*K*): Determined by the algebraic sum of sample audit score. The total audit score is a positive integer that represents degree of untidiness of a given location. An ideal scenario of perfectly clean surface should have an audit score of zero. Using Equation (6) for sample audit score, the total audit score *K* is given by:
  (12) $$K=\sum_{i=1}^{N}S_{2i}$$
  where *N* is the total number of samples collected. The maximum value of total audit score is given by (13):
  (13) $$K_{max}=\sum_{i=1}^{N}S_{2i}^{max}$$
  Since maximum audit score ($S_{2i}^{max}$ ) given by a sample is unity, the maximum possible total audit score $K_{max}$ is *N*.
4. Cleaning benchmark score (Ψ): It is the measure of cleanliness of a surface out of 100. The cleaning benchmark score is determined from Total audit score *K* and *N*, which is given by:
  (14) $$\Psi =\left[1-\frac{K}{K_{max}}\right]\times 100$$
  where *N* is the total number of samples collected and $K_{max}$ is the maximum possible audit score. If the maximum sample audit score (Equation (6)) is 1, $K_{max}$ can be taken as *N*.

## 7. Results and Discussion

The cleaning audit framework is validated through multiple field trials and experiments. Five different locations with different floor types are chosen as the test-bed for the experiment trials. Among the five locations three are indoor and remaining are semi-outdoor environment The robot is operated in each space and allowed to navigate autonomously using the dirt probability exploration strategy.For simulating the condition of dirt accumulation, dust particles are sprinkled in different regions on the experimental location. A mixture of tea dust, bits of paper and crushed dried leaves are taken as materials to simulate the dirt particles. Considering the safety factors, maximum allowed linear velocity of the robot is kept as 0.2 ms${}^{-1}$. After each experiment trails, the 2D map generated by the robot, probable dirt location and the sample points generated are retrieved from the robot. KDE is used to visualize pdf of dirt distribution in the location. An extensive comparative study on the outcome of the proposed system in experiment trials are evaluated.

### 7.1. Experiment Trials

**Figure 8:**
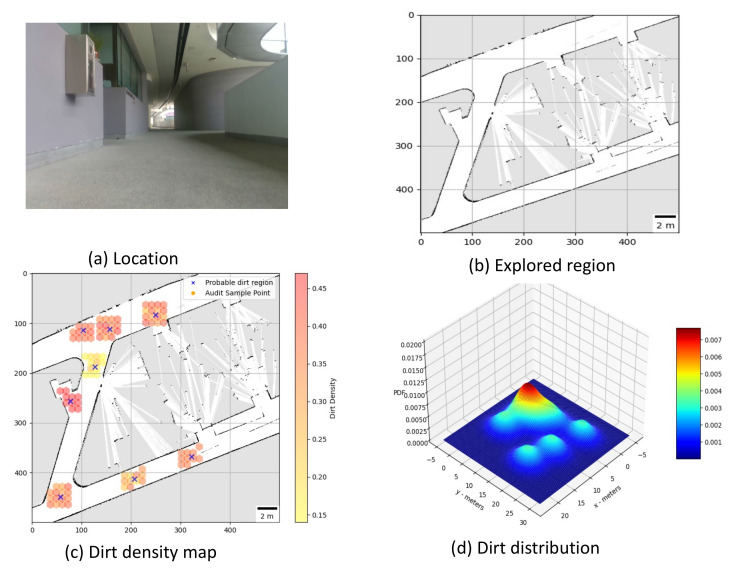
Location under auditing (**a**), Map generated (**b**), audit scores (**c**), dirt distribution (**d**).

**Figure 9:**
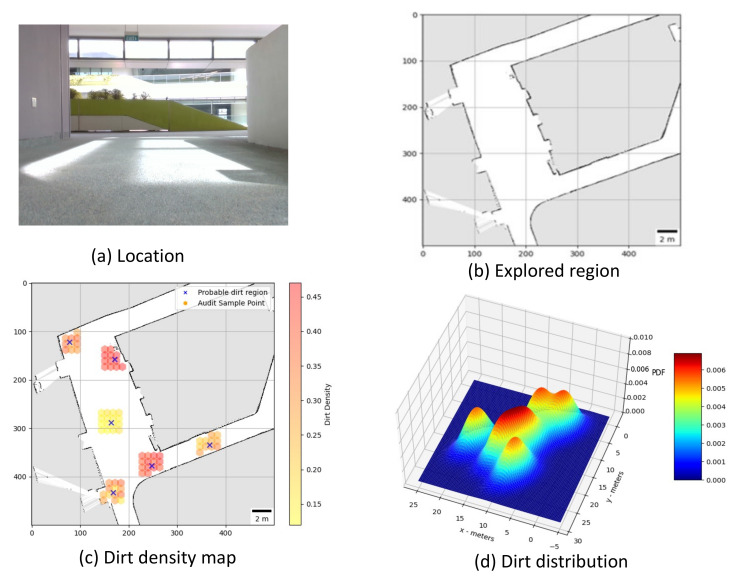
Location under auditing (**a**), Map generated (**b**), audit scores (**c**), dirt distribution (**d**).

**Figure 10:**
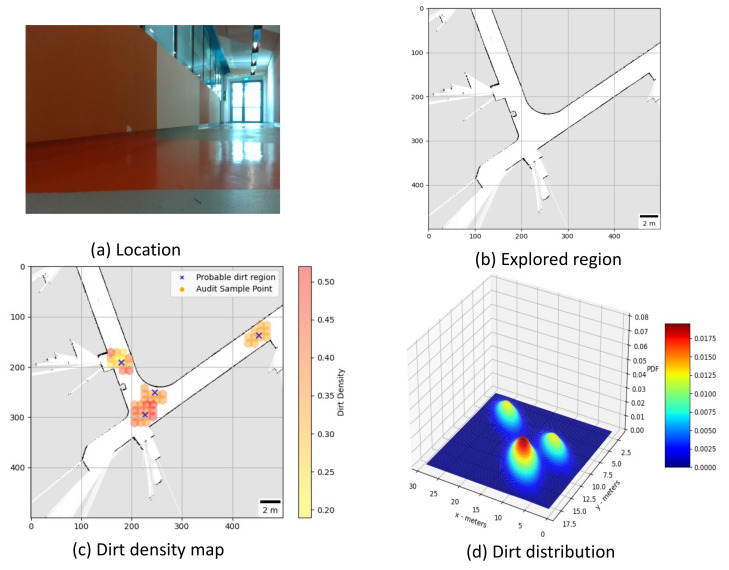
Location under auditing (**a**), Map generated (**b**), audit scores (**c**), dirt distribution (**d**).

**Figure 11:**
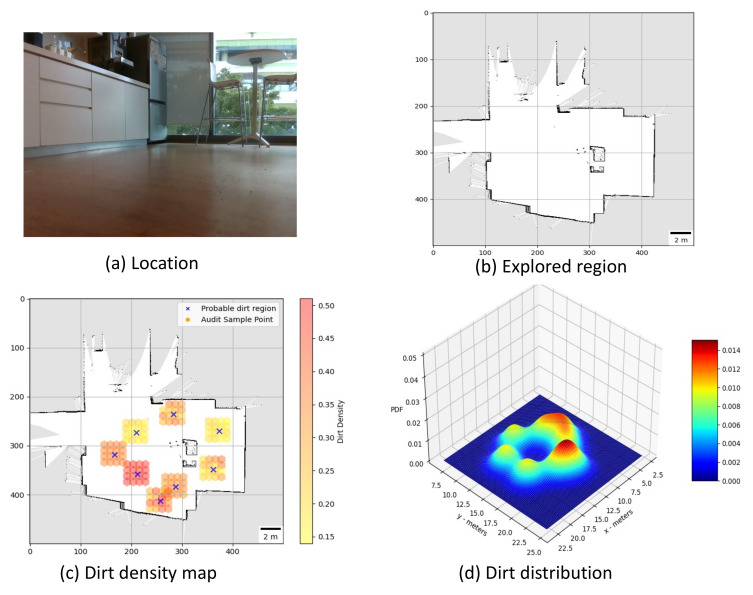
Location under auditing (**a**), Map generated (**b**), audit scores (**c**), dirt distribution (**d**).

**Figure 12:**
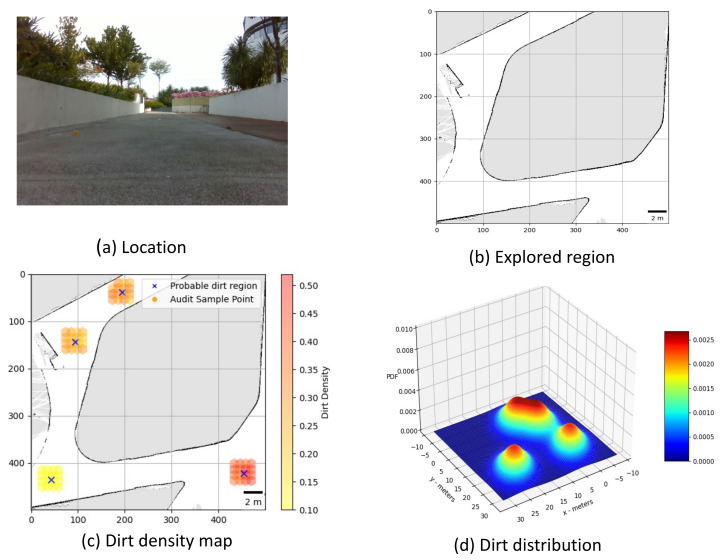
Location under auditing (**a**), Map generated (**b**), audit scores (**c**), dirt distribution (**d**).

1. **Trial 1:** The trial was carried out in an indoor space between the corridor and connecting bridge in the university. The dirt particles are sprinkled in four different locations. Robot identified eight unique probable dirt location corresponds to the identified dirt region. The 2D map of the explored region, location of the sample point, the audit scores corresponds to sample points and the distribution of dirt obtained are given in Figure 8.
2. **Trial 2:** The trial was carried out in a lift lobby. The environment of operation was semi-outdoor with a floor type was coarse cement.Similar to the dirt particles are sprinkled in four different locations. In trial2, Robot identified the dirt locations in six unique probable dirt location. Corresponding to six unique probable dirt location, 76 samples are collected. Even though the number of probable dirt locations are same as trial 1, the positions of the dirt locations identified where close to the map boundaries hence the number of accessible sample points are less compared to trial 1. The 2D map of the explored region, location of the sample point, the audit scores corresponds to sample points and the distribution of dirt obtained are given in Figure 9.
3. **Trial 3:** The trial was carried out in an narrow long indoor corridor. This environment is a comparatively clean area than other trial locations since its an indoor space going under regular maintenance. The floor type was polished vinyl. The dirt particles are sprinkled on four different locations. Robot identified four unique probable dirt location corresponds to the identified dirt region. Robot took total of 48 samples corresponding to the probable dirt locations. The narrow region of the corridor resulted the generation of only 48 sample points which was accessible by the robot.The 2D map of the explored region, location of the sample point, the audit scores corresponds to sample points and the distribution of dirt obtained are given in Figure 10.
4. **Trial 4:** The trial was carried out inside a cafeteria with polished vinyl floor type. The dirt particles where sprinkled in four different locations. Robot identified eight unique probable dirt location corresponds to the identified dirt region. Robot took total of 127 sample points corresponding to probable dirt location. The trial region was spacious compared to the other location this allowed to perform more sampling for inspection The 2D map of the explored region, location of the sample point, the audit scores corresponds to sample points and the distribution of dirt obtained are given in Figure 11.
5. **Trial 5:** The trial was carried out in an semi-outdoor park with coarse cemented and wooden floor type. The dirt particles where sprinkled in four different locations. Robot detected four probable dirt region. A total of 63 sample points are collected.The 2D map of the explored region, location of the sample point, the audit scores corresponds to sample points and the distribution of dirt obtained are given in Figure 12.

### 7.2. Observation and Inference

**Figure 13:**
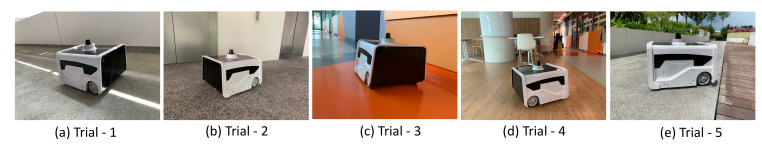
Robot operating on Location 1 (**a**), Location 2 (**b**), Location 3 (**c**), Location 4 (**d**), Location 5 (**e**).

Figure 13 shows the robot’s operation on during the trials and Table 1 consolidates the observation and results obtained from trial–1 to trial–5. The outcome of the trial indicates that, location corresponds to trial–3 got a less audit score compared to other locations. Even though the dust particles are introduced to different trial locations equally, location 3 shows less audit score since, the naturally accumulated dust particles are comparatively low. Since it is an indoor environment having frequent maintenance the possibilities for dirt accumulation is less. The dirt distribution map of location in trial–4 shows a higher variance corresponds to the peeks located around (16.00,10.00) and (5.00,12.50). This pattern corresponds confirms that region where dirt particles accumulated where not far apart and resulted in a rise in average sample a audit score. The significance of higher audit score and dense dirt distribution map shows that, the location is less cleaned, and its cleanliness is lower compared to a similar indoor location with same floor type (location under trial–3). However, the total area explored by the robot and number of samples audited is higher for trial–4. Hence, cleaning benchmark score that provides overall cleaning performance is higher for trial–4. Some of the external factors that influences the accuracy of the framework are:

1. **Floor texture:** The floor with a coarse texture makes the dust particle less susceptible for adhesive dust lifting. However it is acceptable to an extend as long as the cleanliness benchmark is done with similar floor types.
2. **Color of the dust particles:** Some of the dust particles remain undetected during sample auditing, especially dust particles that are more reflective (white paper bits and stapler pins).
3. **Transition between floor types:** The periodic pattern suppression algorithm gives a false positive on detection of probable dirt region when the robot encounters a transition from one floor type to another.

## 8. Conclusions and Future Works

This paper proposes a framework for auditing the cleanliness of built infrastructure using an autonomous mobile robot. The proposed method for cleaning auditing is comprised of sample auditing and space auditing strategies. The sample auditing is accomplished by developing an audit capable of providing audit score of a sample area and space auditing is accomplished using a modified frontier exploration based planning strategy on an in-house developed audit robot with audit sensor on-board. The framework is validated by conducting experiment trials in multiple locations and the insight of dirt distribution has been obtained. The future work of this research will be focusing on:

1. Exploration of electrostatic dirt lifting principle for audit sensor
2. Usage of machine learning based data-driven approach for sample auditing.
3. Improving the sample auditing procedure with odour based sensing.
4. Integration of cleaning audit result to improve the cleaning efficiency of cleaning robots.

## Figures and Tables

**Table 1:** Consolidated results from experiment trials.

	Trial-1	Trial-2	Trial-3	Trial-4	Trial-5
**Environment**	Indoor	Semi-outdoor	Indoor	Indoor	Semi-outdoor
**Floor type**	Polished concrete	Coarse cemented	Vinyl	Vinyl	Cemented and wooden
**Area Explored (m^2^)**	246.70	187.53	118.39	217.11	248.17
**Samples audited**	100	76	48	127	63
**No of dirt locations**	6	6	4	8	4
**Average** **sample audit score**	0.354	0.361	0.376	0.384	0.392
**Peak** **sample audit score**	0.437	0.442	0.495	0.486	0.491
**Peak audit score** **location (m,m)**	(13.00,2.25) (11.15,4.50) (13.50,4.35)	(7.55,8.05) (19.05,12.45) (18.25,13.00)	(13.15,11.00) (15.45,12.05) (14.25,11.15)	(17.45,10.25) (21.00,13.15) (19.35,9.75)	(20.15,21.00) (22.35,22.50)
**Peak dirt distribution** **location (m,m)**	(3,0)	(12,9)	(16,8)	(16,10)	(0,3)
**Exploration time (s)**	1580.0	1551.0	1175.0	1810.0	1247.0
**Total audit score**	37.032	31.152	21.850	48.531	25.739
**Cleaning** **benchmark score** **(Out of 100)**	62.968	59.011	54.479	61.787	59.144

## Data Availability

Data sharing not applicable.
